# Simulation Analysis and Characteristic Research of High-Performance SAW Devices with Trapezoidal Piezoelectric Structures

**DOI:** 10.3390/mi17060705

**Published:** 2026-06-09

**Authors:** Zhipeng Ma, Shijun He, Zhangrui Duan, Lishuang Liu, Jing Zeng, Feng Li

**Affiliations:** School of Electrical and Electronic Engineering, Chongqing University of Technology, No. 69 Hongguang Avenue, Banan District, Chongqing 400054, China; mazhipeng@cqut.edu.cn (Z.M.); 15828594157@163.com (Z.D.);

**Keywords:** electromechanical coupling factor (*K*^2^), surface acoustic wave (SAW), trapezoidal etching, finite-element method (FEM), ZnO/Si structure

## Abstract

The electromechanical coupling factor (*K*^2^) is one of the key parameters characterizing the performance of surface acoustic wave (SAW) devices. Conventional SAW structures suffer from a spatial mismatch between mechanical energy and electric fields, which severely limits improvements in *K*^2^. To address this limitation, this paper proposes a novel microstructure based on trapezoidal etching of the piezoelectric layer. First, an Al/ZnO/Si trapezoidal etching model was established for simulation studies. The results show that trapezoidal etching reduces mechanical energy leakage and enhances the spatial overlap with electric fields. Subsequently, by varying the bottom width (*S*_ZnO_), the variation of *K*^2^ under three etching shapes (standard trapezoidal, rectangular, and inverted trapezoidal) was investigated. The results indicate that trapezoidal etching significantly enhances *K*^2^, which gradually increases as *S*_ZnO_ decreases. Under the theoretical limit (*S*_ZnO_ = 0.1 μm), *K*^2^ reaches a maximum of 14.34%, representing a 19-fold improvement over the conventional structure. Simultaneously, the figure of merit (FOM) and insertion loss (*S*_21_) are also remarkably improved. Finally, considering practical manufacturing constraints, this paper discusses the configurations of *S*_ZnO_ = 0.2 μm and 0.4 μm, revealing that the performance of the SAW devices remains significantly enhanced in both cases, thereby providing a practically feasible solution for the design and fabrication of high-performance SAW devices.

## 1. Introduction

With the rapid development of third-generation semiconductor materials, surface acoustic wave (SAW) devices have been extensively applied in communications [1], automotive electronics [2], aerospace [3], medical diagnostics [4], chemical analysis [5], and sensing applications [6,7,8,9,10]. However, with the advent of the big data era and the increasing complexity of detection environments, high-performance SAW devices featuring a high electromechanical coupling factor (*K*^2^) and low insertion loss (*S*_21_) have garnered significant attention [11]. At the device level, the *K*^2^ is an indispensable parameter for characterizing high-performance SAW devices, as it directly determines their sensitivity and bandwidth. A higher *K*^2^ facilitates greater energy conversion efficiency, faster response speeds, wider bandwidths, and superior sensitivity. Consequently, enhancing *K*^2^ has emerged as a primary research focus for the development of high-performance SAW devices [12].

Currently, doping is a widely adopted method for enhancing *K*^2^. This technique modifies the piezoelectric layer of SAW devices by introducing metal ions, such as V or Al. Fu et al. [13] doped V ions into the ZnO piezoelectric layer of a ZnO/SiC device, increasing the *K*^2^ from 2.8% to 5.12%. Similarly, Iborra et al. [14] compared the piezoelectric properties of pure ZnO and Al-doped ZnO (AZO), finding that AZO exhibited an increase in *K*^2^ from 0.044% to 0.069% compared to conventional ZnO. However, doping methods do not consistently enhance piezoelectric performance and can sometimes lead to a reduction in *K*^2^. For instance, Ralib et al. [15] observed that doping AlN piezoelectric films with V ions did not enhance *K*^2^, but rather reduced it by 1.2% compared to the conventional structure. Alternatively, the superposition method, which involves stacking additional materials onto the piezoelectric layer to form multilayer structures, offers another approach to enhancing *K*^2^. Luo et al. [16] utilized (110)-oriented ZnO to create a three-layer SAW device structure, achieving a high *K*^2^ of 3.37%. Shen et al. [17] proposed a multilayer SAW structure (a-ZnO/a-GaN/r-sapphire) capable of reaching a maximum *K*^2^ of 7%. However, this stacking approach relies heavily on precise material selection and crystal orientation control, which significantly increases manufacturing complexity. In recent years, piezoelectric layer etching has garnered significant attention due to its effectiveness and simplicity. Dun et al. [18] proposed a Ni/etched-ZnO/Si structure using Ni as the conductive material; by etching the piezoelectric layer, the device achieved a *K*^2^ of 4.26%. Furthermore, Dun et al. [19] modified the conductive material to propose a patterned Al/ZnO/Si structure, attaining a maximum *K*^2^ of 7.98%, approximately seven times higher than that of conventional structures. Xu et al. [20] introduced a ZnO/SiC hierarchical structure with etched piezoelectric layers. By optimizing the piezoelectric film thickness, electrode parameters, metallization ratio, and etch ratio, *K*^2^ was increased to 12%, nearly twelve times that of the unetched structure. Fan et al. [21] proposed a Lamb wave resonator with a trapezoidal groove configuration, demonstrating that by adjusting the etching depth, the *K*^2^ could be enhanced to 5.47% without compromising the overall device performance. Similarly, to improve the electromechanical coupling, Fan et al. [22] designed a four-layer IDT/etched-AlN/IDT/AlN/diamond resonator, achieving a *K*^2^ of 3.09%. Furthermore, Liu et al. [23] investigated the variation of *K*^2^ in an AlN/Sapphire structure subjected to trapezoidal etching. Their results revealed that the etched configuration yielded a *K*^2^ of 6.8%, representing a two-fold increase compared to its unetched counterpart. Based on the aforementioned studies, it is evident that although the etching method can effectively enhance the *K*^2^ of SAW devices, this improvement still exhibits certain limitations. Specifically, under the premise of maintaining other optimal device parameters, the maximum reported *K*^2^ is merely limited to 12% [20]. Therefore, there remains substantial room for further optimization of the etching strategy.

To design SAW devices with a high electromechanical coupling factor, this paper utilizes coupling theory to thoroughly analyze the interaction mechanism between mechanical energy and the electric field. Specifically, the analysis focuses on the limitations inherent in conventional Al/ZnO/Si structures, which stem from mechanical energy leakage and the spatial mismatch between the mechanical and electric fields. To address these challenges, a trapezoidal etching structure was proposed and simulated using the finite-element method (FEM). By varying the bottom width (*S*_ZnO_), under Rayleigh mode, the variation characteristics of *K*^2^ and wave velocity (*V*_P_) with the thickness of the piezoelectric material (*H*_ZnO_) and different etching ratios (*d*_ZnO_/*H*_ZnO_) for three etched shapes (standard trapezoid, rectangle, and inverted trapezoid) were systematically investigated. Furthermore, admittance simulations were performed at the point of maximum *K*^2^ to evaluate the corresponding figure of merit (FOM) and *S*_21_, confirming the superior performance of the proposed SAW device design.

## 2. Mechanistic Analysis

The constitutive equation for piezoelectric materials is expressed as [24]:

(1) $$\begin{array}{c} T=c^{E}S-eE\\ D=eS+\varepsilon^{S}E \end{array}$$

where *T* is the stress, *S* is the strain, *E* is the electric field (V/m), *D* is the electric displacement vector, *c^E^* is the elastic stiffness coefficient (N/m^2^), *e* is the piezoelectric constant (C/m^2^), and *ε^S^* is the dielectric permittivity (F/m).

The typical acoustic energy density equation for SAW devices can be provided by Equation (2) [25]:

(2) $$\begin{array}{c} \nu \frac{\partial T}{\partial z}+T\frac{\partial \nu }{\partial z}=\rho \nu \frac{\partial \nu }{\partial t}+T\frac{\partial S}{\partial t} \end{array}$$

where *v* is the particle velocity, *z* is the particle displacement direction, *ρ* is the density, and *t* is the time.

Based on Hooke’s law, substituting Equation (1) into Equation (2) provides the following:

(3) $$\frac{\partial }{\partial z}(-Tv)=\frac{\partial }{\partial t}\left(-\frac{1}{2}\rho v^{2}-\frac{1}{2}c^{E}S^{2}+\frac{1}{2}eES+\frac{1}{2}\varepsilon^{S}E^{2}\right)$$

The electromechanical coupling coefficient serves as a key parameter to evaluate the conversion efficiency between mechanical strain energy and electrical energy in SAW devices. In SAW devices, due to the characteristics of acoustic wave propagation and the constraints of geometric boundaries, the spatial distribution of the strain and electric fields is highly non-uniform. Therefore, taking into account the actual three-dimensional (3-D) (COMSOL Multiphysics 6.2) conditions of SAW devices, the effective electromechanical coupling coefficient can be expressed as the ratio of the square of the electromechanical energy density (*U_me_*) to the product of the mechanical energy density (*U_m_*) and the electrical energy density (*U_e_*):

(4) $$\begin{array}{ll} K^{2} & =\frac{Ume^{2}}{Umc\times Ue}=\left(\frac{e^{2}}{c^{E}\varepsilon^{S}}\right)\times \frac{(\underset{V_{1}}{∭}ESdv{)}^{2}}{(\underset{V_{2}}{∭}S^{2}dv)\times (\underset{V_{3}}{∭}E^{2}dv)}\\ & =\left(\frac{e^{2}}{c^{E}\varepsilon^{S}}\right)\times \Gamma \end{array}$$

where *V* is the effective volume and Γ is the coupling factor.

As indicated by Equation (4), the key to enhancing the electromechanical coupling coefficient lies in increasing the coupling factor Γ. However, since the electric field and mechanical strain are mutually coupled within Γ, increasing the numerator inevitably leads to a simultaneous increase in the denominator, and vice versa. Therefore, the magnitude of the electromechanical coupling coefficient is not determined merely by the absolute values of the physical fields or material parameters, but rather by the effective spatial coupling between the electrical and mechanical energies within the SAW device. This coupling effect can be clearly demonstrated through numerical simulations.

The model of the SAW device is governed by Maxwell’s equations, Newton’s laws, the piezoelectric equation, and Gauss’s law, as shown in (5) [26,27,28]:

(5) $$\begin{array}{l} \sum_{j,k,l}c_{ijkl}\frac{\partial^{2}u_{j}}{\partial x_{j}\partial x_{i}}+\sum_{j,k}e_{kij}\frac{\partial^{2}E}{\partial x_{j}\partial x_{k}}=\rho \frac{\partial^{2}u_{i}}{\partial^{2}t}\\ \sum_{k,l}e_{ikl}\frac{\partial^{2}u_{l}}{\partial x_{j}\partial x_{k}}=\sum_{k}\varepsilon_{ik}\frac{\partial^{2}E}{\partial x_{i}\partial x_{k}} \end{array}$$

where *u_i_* is the particle displacement of the piezoelectric layer, *E* is the electric field vector (V/m), *c_ijkl_* is the elastic constant tensor (N/m^2^), *ε_ik_* is the permittivity tensor (F/m), and *e_ikl_* is the piezoelectric constant tensor (C/m^2^).

**Figure 1:**
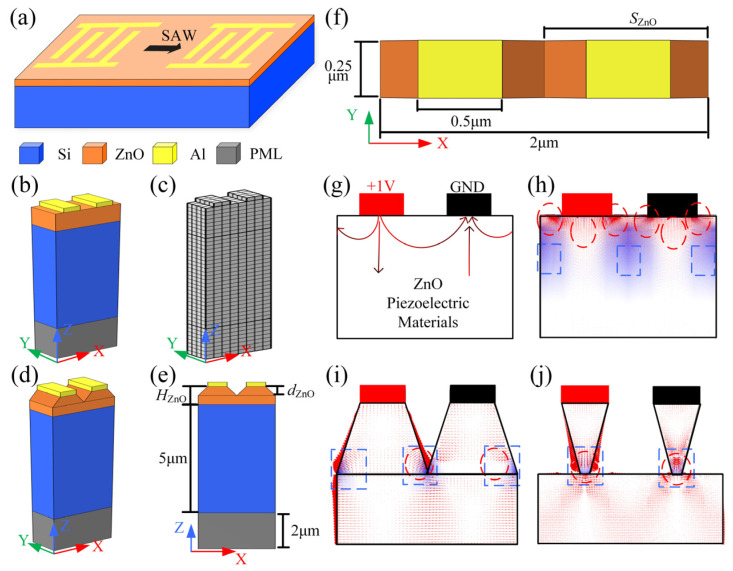
Illustration of model construction and energy distributions in the FEM simulation. (**a**) Overall structure of the SAW device. (**b**) Conventional structural model. (**c**) Mesh generation of the conventional structure model. (**d**) Trapezoidally etched model (*S*_ZnO_ = 1 μm). (**e**,**f**) Front and top view of the trapezoidally etched model. (**g**) Actual electric field distribution. (**h**) Distributions of the electric field and mechanical energy in the conventional structure. (**i**,**j**) Distributions of the electric field and mechanical energy in the trapezoidally etched model.

Based on the governing equations in (5), the simulation model of the SAW device was established by using the FEM. The structure primarily consists of four domains: the Si substrate, the ZnO piezoelectric layer, the Al interdigitated transducer (IDT) electrodes, and a Si perfectly matched layer (PML) to suppress boundary reflections, as shown in Figure 1a. The IDT layer comprises two sets of periodically arranged comb-like metallic strip electrodes, which serve to excite and detect the surface acoustic waves. Leveraging the structural periodicity of the device, a single period was extracted to construct the conventional Al/ZnO/Si model, as shown in Figure 1b [29]. The simulation parameters are defined with the wavelength (*λ* = 2 μm). The geometric dimensions are set as follows: Al electrodes (0.5 μm × 0.25 μm × 0.25 μm), Si substrate (2 μm × 0.25 μm × 8 μm), and the ZnO layer thickness of 1 μm. Corresponding periodic boundary conditions are applied. Although a finer mesh provides higher simulation accuracy, it also leads to an exponential increase in data volume and computational workload. Therefore, to achieve an optimal balance between accuracy and computational cost, a minimum mesh size of λ/8 was adopted in this study. The mesh partitioning diagram is shown in Figure 1c. Similarly, while keeping the basic dimensional parameters and materials unchanged, the piezoelectric layer was etched to vary the etching ratio (*d*_ZnO_/*H*_ZnO_) and the bottom width (*S*_ZnO_). This yielded a simulation model of the Al/ZnO/Si structure with a trapezoidal profile, as shown in Figure 1d. Figure 1e,f illustrate the front and top views of the trapezoidally etched model, respectively.

The intensity and direction of the electric field are shown by the density of the field lines. Figure 1g displays the actual electric field distribution under an applied IDT voltage, while Figure 1h maps the mechanical energy (blue regions) and electric field (red lines) in the conventional structure. The simulations reveal a highly non-uniform electric field that is primarily confined to the top of the piezoelectric layer. Conversely, the mechanical energy fails to maintain this confinement, exhibiting significant acoustic leakage into the surrounding and underlying regions. This dissipated mechanical energy couples only weakly with the electric field, fundamentally hindering the enhancement of the electromechanical coupling coefficient.

To tackle this energy leakage, a trapezoidal etching strategy is applied to the piezoelectric layer to cut off the diffusion channels. This geometrically confines the mechanical energy strictly to the top region, allowing it to perfectly coincide with the densest electric field. As shown by the simulated distributions in Figure 1i,j, the etched models maintain an electric field profile similar to the conventional one, yet successfully eliminate the massive mechanical diffusion. By tightly concentrating both fields at the top surface, this structure achieves deep spatial overlap and full electromechanical coupling, thereby maximizing the coupling coefficient.

To quantitatively analyze the trapezoidally etched structure, based on equations in (4), the *K*^2^ is directly proportional to the Γ, which can be expressed as follows:

(6) $$K^{2}\propto \Gamma =A^{2}/(B\times C)$$

**Figure 2:**
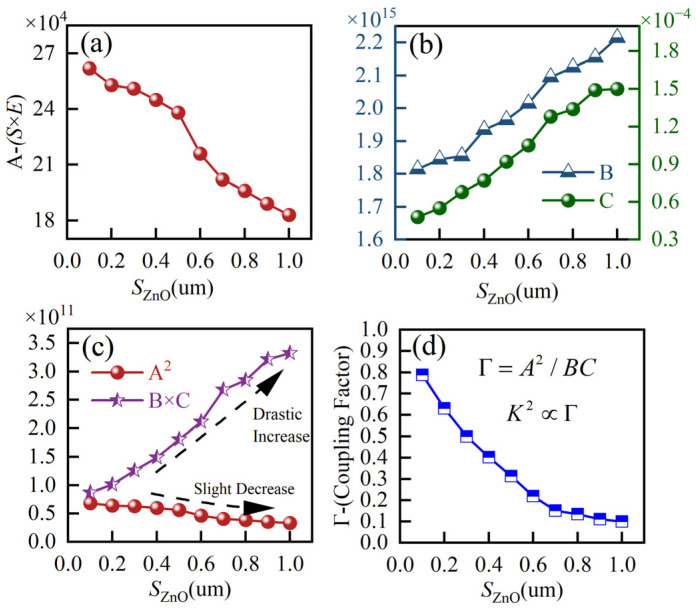
Variations of the mechanical strain energy and electrical energy in the trapezoidally etched model as a function of *S*_ZnO_. (**a**) Electromechanical energy density A. (**b**) Electrical energy density B and mechanical strain energy density C. (**c**) The calculated A^2^/(B × C). (**d**) The coupling factor Γ.

With the increase in *S*_ZnO_, the enlarged diffusion of mechanical energy weakens the electromechanical coupling effect, leading to a pronounced drop in the electromechanical energy density (A). In contrast, both the electrical energy density (B) and the mechanical energy density (C) exhibit a gradual increase, as shown in Figure 2a,b. Figure 2c,d plot the evolution of the mathematical term A^2^/(B × C) and the overall coupling factor Γ. Notably, driven by the overwhelming magnitude of (B × C) relative to A^2^, the coupling factor Γ strictly decreases as *S*_ZnO_ widens, thereby dictating a proportional decay in the macroscopic coupling coefficient *K*^2^.

**Figure 3:**
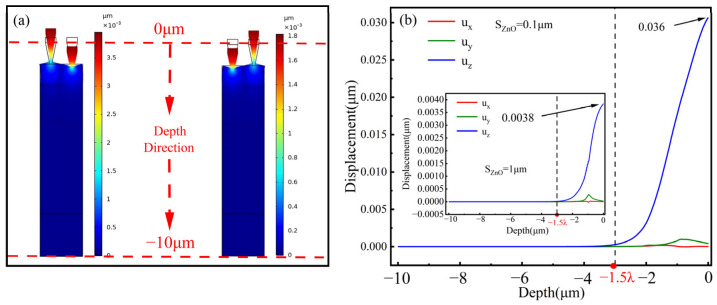
FEM-simulated vibration mode and displacement of the trapezoidally etched structure. (**a**) Vibration mode. (**b**) Displacement distribution.

Figure 3 shows the FEM-simulated vibration mode and displacement profiles of the trapezoidally etched SAW device. As shown in Figure 3a, the vibration amplitude decays with the depth of the piezoelectric layer. By defining the surface of the piezoelectric material as the reference zero point, the variation of the acoustic wave displacement along the depth is shown in Figure 3b. The displacement components along the X-, Y-, and Z-direction are denoted as *u_x_*, *u_y_*, and *u_z_*, respectively. It can be observed that the *u_x_* and *u_y_* components exhibit negligible displacement. The *u_z_* component dominates the displacement and is primarily concentrated within a depth of approximately 3 μm (1.5*λ*) from the surface. At depths exceeding 1.5*λ*, the *u_z_* displacement approaches zero. These results confirm that the acoustic waves generated by the etched SAW device exhibit characteristic Rayleigh wave behavior.

The wave velocity (*V*_P_) of Rayleigh waves is calculated as follows [30,31]:

(7) $$V_{P}=\lambda \frac{f_{r}+f_{\mathrm{ar}}}{2}$$

Similarly, the *K*^2^ of Rayleigh waves is calculated as follows:

(8) $$K^{2}=\frac{\pi f_{r}}{2f_{\mathrm{ar}}}/\tan\left(\frac{\pi f_{r}}{2f_{\mathrm{ar}}}\right)$$

where *f*_r_ is the resonant frequency and *f*_ar_ is the anti-resonant frequency.

**Figure 4:**
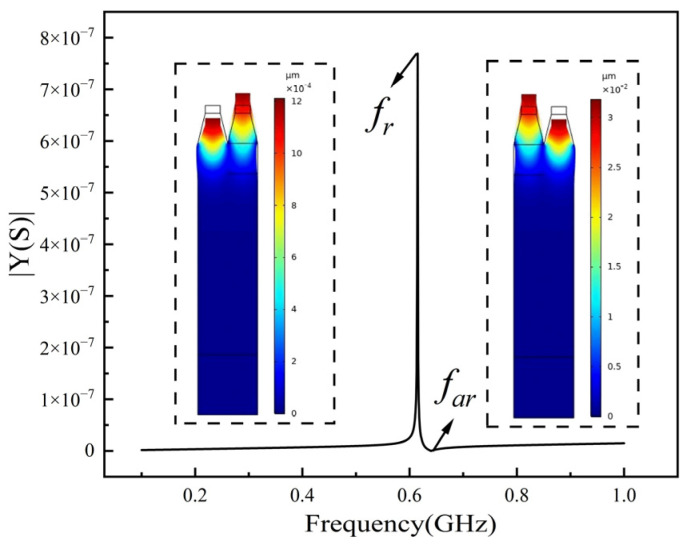
Calculated admittance curve of the Rayleigh wave (*S*_ZnO_ = 0.1 μm).

These parameters are obtained from the Rayleigh wave conductance curve, as shown in Figure 4. On the admittance curve (|Y(S)|), *f*_r_ is the maximum value near the operating frequency, while *f*_ar_ is the minimum value.

The SAW generated by the trapezoidal etching of the Al/ZnO/Si structure modifies the conventional mechanism, in which piezoelectric materials directly excite SAW. This approach accounts for the coupling effect at the structural boundaries and incorporates process optimization of the structural dimensions through etching. Therefore, the influence of structural dimensions (*H*_ZnO_, *d*_ZnO_/*H*_ZnO_, *S*_ZnO_) on the electromechanical characteristics of SAW devices is investigated.

## 3. Results and Discussion

For trapezoidal etching, different etching shapes appear as *S*_ZnO_ decreases. Specifically, when 0.5 μm < *S*_ZnO_ ≤ 1 μm, the shape assumes a normal trapezoid; when *S*_ZnO_ = 0.5 μm, it takes the form of a rectangle; and when 0.1 μm ≤ *S*_ZnO_ < 0.5 μm, it transforms into an inverted trapezoid. This study investigates how the SAW device parameters, *K*^2^ and *V*_P_, vary with the normalized thickness (*H*_ZnO_/λ) under different etching ratios for these three etching shapes.

**Figure 5:**
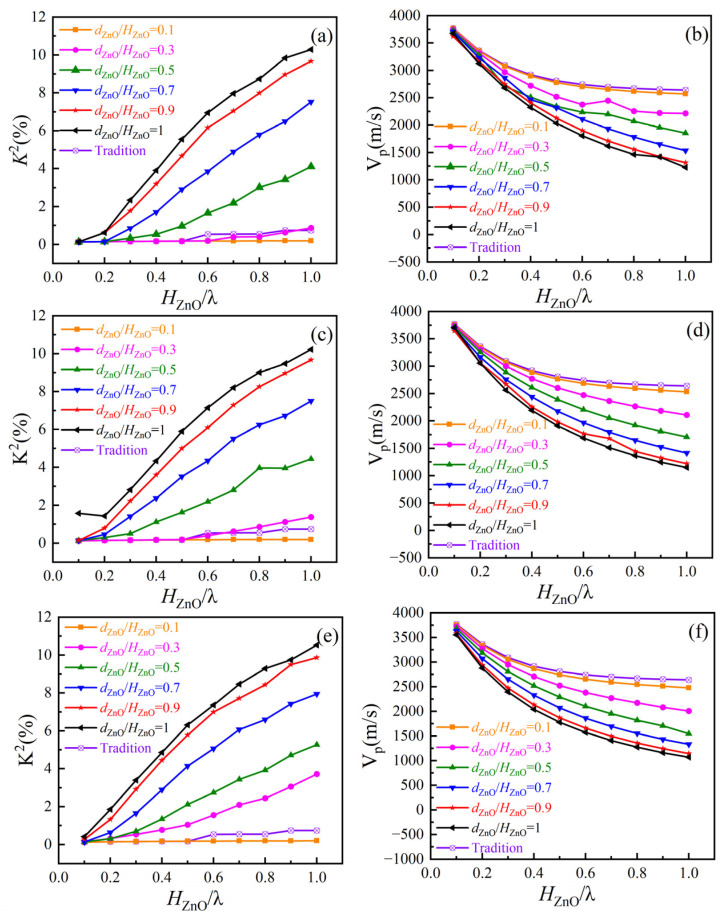
Simulation results showing the variation of *K*^2^ and *V*_P_ for the standard trapezoidally etched Al/ZnO/Si structure. (**a**,**b**) *S*_ZnO_ = 1 μm. (**c**,**d**) *S*_ZnO_ = 0.8 μm. (**e**,**f**) *S*_ZnO_ = 0.6 μm.

Figure 5 shows the variation of *K*^2^ and *V*_P_ with normalized thickness for SAW devices under different etching ratios during normal trapezoidal etching. As shown in Figure 5a,c,e, when *d*_ZnO_/*H*_ZnO_ = 0.1–0.3, the increase in *K*^2^ is relatively slow compared to the conventional structure, and *K*^2^ can even be lower than that of the conventional structure, likely due to the negative effect of weak etching [27]. The effect of etching becomes significant at *d*_ZnO_/*H*_ZnO_ = 0.5, resulting in a notable increase in *K*^2^ from 0.742%. When *d*_ZnO_/*H*_ZnO_ = 1 and *H*_ZnO_/λ = 1, the *K*^2^ of the SAW device reaches its maximum value. The energy of the Rayleigh wave is primarily concentrated within a depth of 1λ to 2λ from the surface. Once the piezoelectric material thickness reaches 1λ, it maximally prevents the leakage of Rayleigh wave energy into the non-piezoelectric substrate (Si). Simultaneously, the trapezoidal etching technique eliminates lateral energy dispersion channels, enabling optimal spatial coupling between mechanical energy and the electric field. This conclusion is consistent with [19,20,30]. At this optimal point, the maximum *K*^2^ is 10.522%, corresponding to an increase of 14.18 times relative to the conventional structure. Meanwhile, *V*_P_ reaches its minimum value of 995.1 m/s when *K*^2^ is maximized, as shown in Figure 5b,d,f.

**Figure 6:**
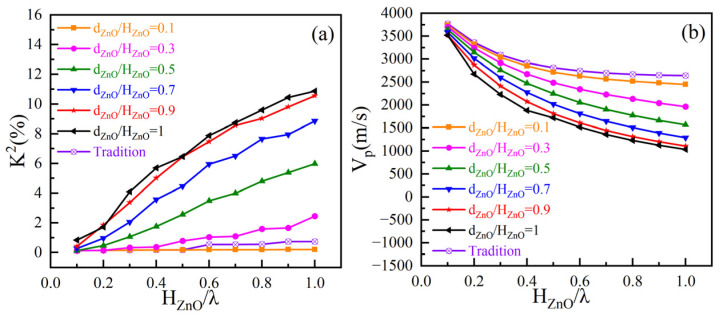
Simulated variation for the rectangularly etched Al/ZnO/Si structure. (**a**) *K*^2^. (**b**) *V*_P_.

Figure 6 shows the simulated variation of *K*^2^ and *V*_P_ with normalized thickness for SAW devices under different etching ratios during rectangular etching. The variation trends of *K*^2^ and *V*_P_ for rectangular etching are similar to those of normal trapezoidal etching. Under the condition that *S*_ZnO_ is fixed, *d*_ZnO_/*H*_ZnO_ = 1, and *H*_ZnO_/λ = 1, *K*^2^ reaches its maximum value of 10.875%, corresponding to an increase of 14.65 times relative to the conventional structure. At this point, *V*_P_ reaches its minimum value of 1034.4 m/s.

**Figure 7:**
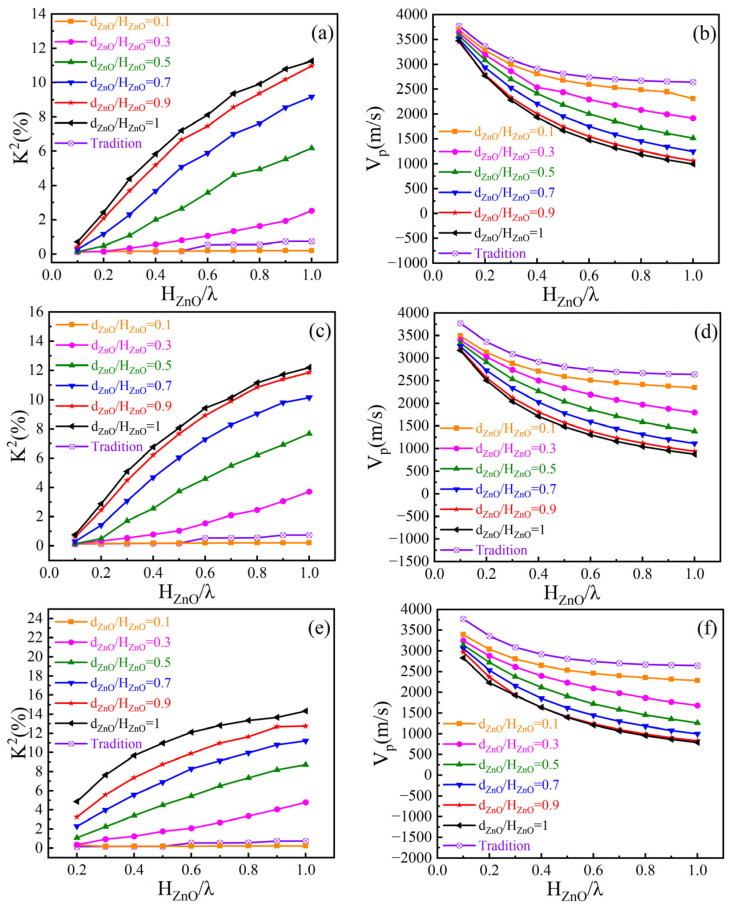
Simulated variation of *K*^2^ and *V*_P_ for the inverted trapezoidally etched Al/ZnO/Si structure. (**a**,**b**) *S*_ZnO_ = 0.4 μm. (**c**,**d**) *S*_ZnO_ = 0.2 μm. (**e**,**f**) *S*_ZnO_ = 0.1 μm.

Similarly, as *S*_ZnO_ decreases below 0.5 μm, the etching shape becomes an inverted trapezoid. Figure 7 shows the variation of *K*^2^ and *V*_P_ with normalized thickness for SAW devices under different etching ratios during inverted trapezoidal etching. As shown in Figure 7a,c,e, when *S*_ZnO_ = 0.1 μm, *d*_ZnO_/*H*_ZnO_ = 1, and *H*_ZnO_/λ =1, the SAW device reaches its maximum *K*^2^ of 14.34%, which corresponds to an increase of more than 19 times relative to the conventional structure. In summary, when *S*_ZnO_ is fixed, *K*^2^ increases with normalized thickness, and as the piezoelectric etching ratio increases, *K*^2^ gradually approaches its maximum, while *V*_P_ decreases correspondingly.

**Figure 8:**
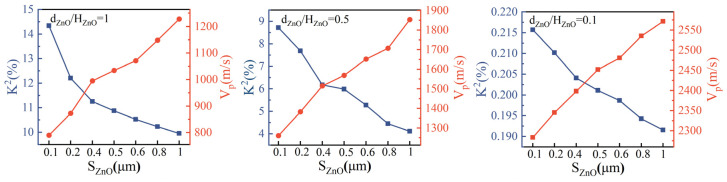
Variation of *K*^2^ and *V*_P_ with bottom width (*S*_ZnO_) for the SAW devices (*H*_ZnO_/λ = 1).

To investigate the effect of *S*_ZnO_ on *K*^2^, the conditions *d*_ZnO_/*H*_ZnO_ = 1 and *H*_ZnO_/λ =1 were selected. The simulated variation of *K*^2^ and *V*_P_ with bottom width (*S*_ZnO_) for the SAW devices is shown in Figure 8. The results indicate that *K*^2^ decreases with increasing *S*_ZnO_, with a maximum value of 14.34%. Conversely, *V*_P_ increases as *K*^2^ decreases, exhibiting an opposite trend. To confirm the correctness of this behavior, additional simulations were conducted for *d*_ZnO_/*H*_ZnO_ = 0.5 and *d*_ZnO_/*H*_ZnO_ = 0.1. The results shown that the variation patterns of *K*^2^ and *V*_P_ remained consistent across all conditions.

The preceding analysis reveals that the trapezoidal etched structure enables SAW devices to achieve a high *K*^2^, thereby delivering significant performance improvements. However, the high performance of a SAW device is not solely reflected by the *K*^2^ parameter alone. In practice, an analysis combining the FOM and *S*_21_ is typically used as the standard for evaluating device performance. Therefore, while achieving a high *K*^2^, it is essential to simultaneously investigate the FOM and *S*_21_ [29,30].

**Figure 9:**
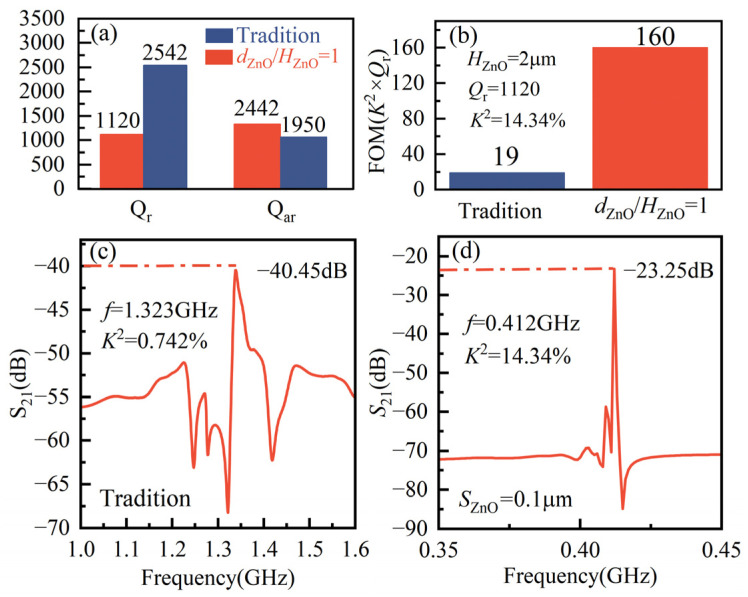
Variations in the FOM and *S*_21_ of the SAW devices. (**a**) Resonance (*Q*_r_) and antiresonance (*Q*_ar_) quality factors. (**b**) FOM. (**c**) *S*_21_ of the conventional structure. (**d**) *S*_21_ of the trapezoidally etched structure.

Figure 9 shows the variation of the FOM and *S*_21_ for the SAW device at maximum *K*^2^. To accurately evaluate the FOM of the SAW device (FOM = *K*^2^ × *Q*_r_), the −3 dB bandwidth method was employed to extract the resonance quality factor (*Q*_r_) and anti-resonance quality factor (*Q*_ar_) [31], as depicted in Figure 9a. It can be observed that *Q*_ar_ increases significantly from 1950 to 2442, whereas *Q*_r_ decreases from 2542 to 1120. This reduction in *Q*_r_ is likely attributed to the increased electrical resistance caused by the narrowed piezoelectric layer after etching, as well as the emergence of spurious modes [32,33,34,35]. Nevertheless, compared to the conventional structure, the overall FOM of the etched SAW device is substantially enhanced, increasing from 19 to 160, as shown in Figure 9b. Similarly, the *S*_21_ is significantly improved following the trapezoidal etching; near the operating frequency, *S*_21_ reaches an optimal value of −23.25 dB, representing a 42.5% reduction in absolute insertion loss compared to the conventional model, as shown in Figure 9c,d. This improvement is primarily attributed to the strong spatial coupling between the mechanical energy and the electric field induced by the trapezoidal etching, which effectively mitigates energy leakage [18].

## 4. Fabrication Trade-Offs

It is worth noting that although the performance of the SAW device is significantly enhanced when *K*^2^ is maximized, the extreme profile (*S*_ZnO_ = 0.1 μm) associated with this optimal inverted trapezoidal structure renders the device susceptible to severe microfabrication challenges and mechanical fracture risks. Among the three etching topologies investigated in this study (standard trapezoidal, rectangular, and inverted trapezoidal), the standard trapezoidal and rectangular geometries can effectively circumvent the aforementioned risks [19,21]. however, their enhancement of the *K*^2^ remains limited. Therefore, despite the inherent fabrication risks of the inverted trapezoidal profile, systematically investigating the performance of SAW devices under less extreme inverted trapezoidal conditions (*S*_ZnO_ = 0.2 μm and *S*_ZnO_ = 0.4 μm) remains highly instructive for practical engineering applications. This optimal trade-off ensures that the proposed design possesses high engineering viability under current fabrication capabilities.

**Figure 10:**
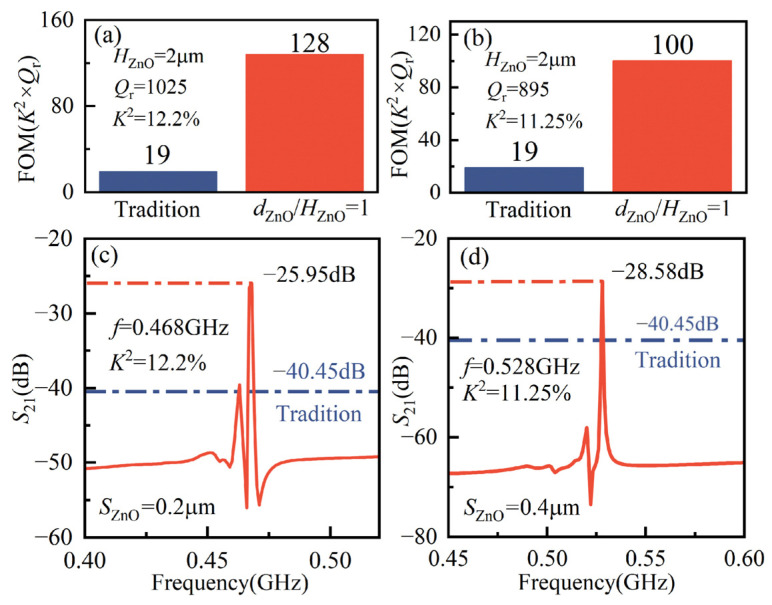
Variations in FOM and *S*_21_ under different inverted trapezoidal etching conditions. (**a**,**c**) *S*_ZnO_ = 0.2 μm. (**b**,**d**) *S*_ZnO_ = 0.4 μm.

Figure 10 shows the variations in the figure of merit (FOM) and *S*_21_ of the SAW devices under two inverted trapezoidal etching conditions (*S*_ZnO_ = 0.2 μm and *S*_ZnO_ = 0.4 μm). When *S*_ZnO_ = 0.2 μm, the FOM increases to 128 and the *S*_21_ improves to −25.95 dB, as depicted in Figure 10a,c. It is noteworthy that the *S*_ZnO_ = 0.4 μm configuration also significantly enhances device performance; although the improvement is less pronounced than that of the 0.2 µm case, the FOM still rises from 19 to 100, and the *S*_21_ improves from −40.45 dB to −28.58 dB (representing a 29.34% reduction in *S*_21_), as shown in Figure 10b,d. These results indicate that while the optimal performance is achieved at *S*_ZnO_ = 0.1 μm, the *S*_ZnO_ = 0.2 μm and *S*_ZnO_ = 0.4 μm profiles remain highly effective. Crucially, these configurations successfully balance manufacturing feasibility with the mitigation of mechanical fracture risks, offering a robust technical route for the development of high-performance SAW devices.

## 5. Conclusions

Through theoretical analysis, this paper identifies the critical issues inherent in the conventional model, specifically severe mechanical energy leakage and its spatial mismatch with the electric field. To address these limitations, a trapezoidally etched Al/ZnO/Si structure is proposed. Compared with the conventional model, this structure effectively confines mechanical energy and optimizes its coupling with the electric field. Furthermore, the investigation into the influence of the piezoelectric etching geometry demonstrates that optimizing the etching parameters significantly enhances the *K*^2^ and the FOM, while also yielding a marked improvement in *S*_21_. Furthermore, this study also investigates the process trade-off configurations of *S*_ZnO_ = 0.2 μm and 0.4 μm. Even when considering practical manufacturing constraints, these configurations still guarantee a significant enhancement in the electromechanical coupling factor of the SAW devices, thereby providing a valuable reference for the fabrication of high-performance SAW devices and demonstrating highly promising application prospects.

## Data Availability

The original contributions presented in this study are included in the article. Further inquiries can be directed to the corresponding author.
